# A Review of Dietary Selenium Intake and Selenium Status in Europe and the Middle East

**DOI:** 10.3390/nu7031494

**Published:** 2015-02-27

**Authors:** Rita Stoffaneller, Nancy L. Morse

**Affiliations:** 1 49 Station Road, Polegate, East Sussex, BN26 6EA, UK; E-Mail: rita.stoffaneller@outlook.com; 2 Research consultant to Wassen International Ltd. Cedar Court Office Park, Denby Dale Road, Wakefield WF4 3DB, UK; 3 9 Horsburgh Dr., Berwick, N.S., B0P 1E0, Canada

**Keywords:** selenium, dietary intake, nutrient status, Europe, Middle East, United Kingdom, deficiency

## Abstract

This is a systematic review of existing data on dietary selenium (Se) intake and status for various population groups in Europe (including the United Kingdom (UK)) and the Middle East. It includes English language systematic reviews, meta-analyses, randomised controlled trials, cohort studies, cross-sectional and case-control studies obtained through PUBMED searches from January, 2002, to November, 2014, for European data and from 1990 to November 2014, for Middle Eastern data. Reports were selected if they included data on Se intake and status. The search identified 19 European/UK studies and 15 investigations in the Middle East that reported Se intake and Se concentration in water and/or food and 48 European/UK studies and 44 investigations in the Middle East reporting Se status. Suboptimal Se status was reported to be widespread throughout Europe, the UK and the Middle East, and these results agreed with previous reports highlighting the problem. Eastern European countries had lower Se intake than Western European countries. Middle Eastern studies provided varying results, possibly due to varying food habits and imports in different regions and within differing socioeconomic groups. In conclusion, Se intake and status is suboptimal in European and Middle Eastern countries, with less consistency in the Middle East.

## 1. Introduction

Interest in selenium (Se) has been growing over the past few decades in a number of areas of human health. The nutritional status of this metalloid has been difficult to assess via food intake data alone, because many factors influence its presence in the food chain. Although its distribution in soil is uneven [1] , a number of other factors affect its concentration in various foods, including varying uptake into plants due to soil pH, rainfall, land contour and microbial activity [2] and importation of food from higher Se areas. Therefore, estimates of Se intake from food nutrient databases alone can give inaccurate measures of Se status within a population or population groups.

Se is an essential non-metal trace element [3] that is required for selenocysteine synthesis and is essential for the production of selenoproteins [4]. Selenoproteins are primarily either structural or enzymatic [2], acting as catalysts for the activation of thyroid hormone and as antioxidants, such as glutathione peroxidases (GPxs) [5]. GPx activity is commonly used as a marker for Se sufficiency in the body [6], where serum or plasma Se concentrations are believed to achieve maximum GPx expression at 90–100 μg/L (90.01 μg/L as proposed by Duffield and colleagues [7] and 98.7 μg/L according to Alfthan *et al.* [8]). However, plasma selenoprotein P (SEPP1) concentration is a more suitable marker than plasma GPx activity [9]. Prospective studies provide some evidence that adequate Se status may reduce the risk of some cancers, while elevated risk of type 2 diabetes and some cancers occurs when the Se concentration exceeds 120 μg/L [10]. Higher Se status has been linked to enhanced immune competence with better outcomes for cancer, viral infections, including HIV progression to AIDS, male infertility, pregnancy, cardiovascular disease, mood disorders [2] and, possibly, bone health [11,12,13,14].

**Figure 1:**
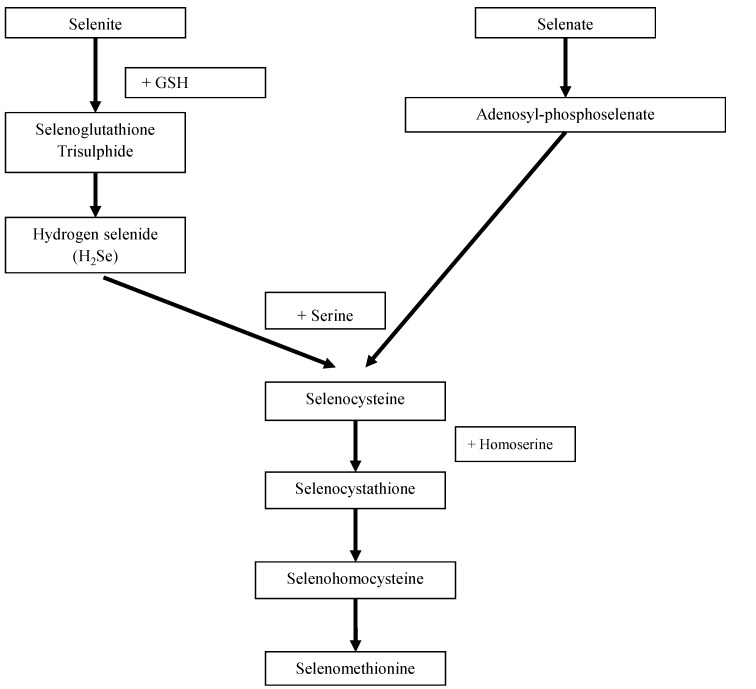
Biosynthetic pathway of selenocysteine and selenomethionine in plants [3].

The two main dietary forms of Se are selenocysteine derived from animal-sourced foods and selenomethionine obtained from animal-sourced foods and cereal products grown on Se-rich soil in areas, such as in the United States (US) [6]. However, animal-derived foods tend to be a better dietary source of this nutrient for humans, because Se is required as an essential nutrient for animal life, and animal feed is sometimes supplemented with Se [15]. Figure 1 shows the biosynthetic pathway for these two amino acids in plants, marine algae and brewer’s yeast.

Health conditions caused by severe Se deficiency include Keshan disease and Kashin-Beck disease. However, both of these conditions occur more often in conjunction with iodine deficiency or in the presence of certain environmental toxins. In the case of Keshan disease, viral infection has also been implicated [2].

The Food and Nutrition Board at the Institute of Medicine of the National Academies, US, has recommended, for 19–50-year-old men and women, 45 micrograms (μg) of Se/day as the estimated average requirement (EAR), 55 μg of Se/day as the recommended dietary allowance (RDA) and 400 μg of Se/day as the tolerable upper intake level (UL) [4,16]. A peer-reviewed article quoted a safety intake recommendation at around 800 μg of Se/day for no observed adverse effect level (NOAEL), 1540 to 1600 μg of Se/day for low observed adverse effect level (LOAEL) and 5000 μg of Se/day for a toxic level, where selenosis occurs [17]. LOAEL symptoms have been associated with hair, toe and fingernail loss and a garlicky odour on the breath, whilst toxicity can cause acute respiratory distress syndrome, myocardial infarction and renal failure [4].

The recommended reference nutrient intake (RNI) of Se for adults in the United Kingdom (UK) is 60 μg/day for adult women and 75 μg/day for lactating women and adult men [18]. The US daily recommended intake (DRI) is 55 μg/day for both men and women [16] and the European Food Safety Authority (EFSA), in the European Union (EU), has recently set a daily adequate intake for Se at 70 μg [9]. To date, the optimal intake to achieve additional health benefits over general health maintenance and the best biological marker(s) to assess Se sufficiency have not been conclusively established [3].

It is generally recognised that Se intakes across Europe are low, reflecting inadequate soil levels, particularly in Eastern Europe. Se intake in the UK has declined since the 1970s, and previous government surveys [19] indicate low Se intake across a wide age range of the UK population (Caucasian and Asian) [20].

A number of studies investigating Se levels in blood (serum and/or plasma), breast milk and umbilical cord blood indicate generally low Se status in women from the Kingdom of Saudi Arabia (KSA), Kuwait and Turkey [21,22,23], but to date, to the author’s knowledge, no systematic reviews have been completed on the data originating from the Middle East. In 2002, Rayman *et al.* [2] reported similarly low serum or plasma Se status in numerous European countries. In this publication, we review and summarize Se intake and status data collected and reported since 2002 in European countries and since 1990 for Middle Eastern countries. A comparison of the overall Se status in the UK and Middle Eastern countries was of interest to the authors, since a manuscript pertaining to Se status in Saudi Arabian women and UK age-matched controls and its influence on calcium homeostasis, bone density and skeletal metabolism [24] was in preparation.

## 2. Experimental Section

A review of current literature was undertaken to obtain data on Se intake and status in European and Middle Eastern countries. A review article containing relevant data pertaining to European countries had been previously published in 2002 [2]. Therefore, 2002 was used as the starting point for studies selected from the literature search for inclusion in this systematic review. However, there have been no previously published review articles on the subject for Middle Eastern countries. Rather than completing an extensive historical search, the year 1990 was selected as a starting point for Middle Eastern studies to include in this systematic review.

A systematic search was performed on PUBMED for English-language articles published from January, 2002, through to November, 2014, to obtain European data and from January, 1990, through to November, 2014, to obtain Middle Eastern data using the following search words: bone health and Middle East/KSA/UK, dietary surveys and Middle East/KSA/UK, selenium and bone, antioxidants and bone, selenium gene expression and bone, selenium status and Middle East/KSA/UK, selenium levels in food and water, vitamin D/vitamin D status and bone/Middle East/KSA/UK, vitamin D and selenium dependent enzymes, vitamin D and osteoporosis and vitamin D deficiency and osteoporosis. Additional studies were identified through a Google search using the search criterion, selenium deficiency. Titles and abstracts were reviewed and reports were selected for inclusion in the review if they were systematic reviews, meta-analyses, randomised controlled trials, cohort studies, cross-sectional or case-control studies and if they included data on Se intake and status.

## 3. Results

### 3.1. Selenium Intake Studies

The literature search identified 19 European/UK studies and 15 investigations in the Middle East that reported Se intake and Se concentration in water and/or food. A summary of the findings are presented in Table 1 and Table 2.

#### 3.1.1. Selenium Intake Studies: European Countries/UK

Eastern European countries tended to have lower Se intake than their western counterparts, as indicated by a Polish study [25], where Se content of the consumed foods was four-times lower than in Spain, which tended to have intakes that exceeded the DRI and RDA [26,27,28,29,30]. Studies in France [31] and Belgium [32] reported intakes equivalent to the RDA, while those from Slovenia [33] and Italy [34,35] found intakes below the RDA.

**Table 1:** Studies investigating Se intake and Se concentration in water and food in Europe (including the UK). RNI, reference nutrient intake; DRI, daily recommended intake; LRNI, lower RNI.

Country	Study	Subject No./Age/Samples Details	Se Intake/Water and Food Content
UK [20]	A longitudinal analysis of Se intakes in UK South Asian and Caucasian women	135 Caucasian premenopausal (19–40 years), 135 Caucasian postmenopausal (55–70 years), 39 Asian premenopausal (19–40 years) and 39 Asian postmenopausal (55–70 years) women	Se intake in a large proportion of subjects was less than the UK RNI (60 μg/day), the US DRI (55 μg/day) and UK LRNI (40 μg/day). Most women (all ethnicities) failed to meet UK RNI, US DRI and UK LRNI respectively
Poland [25]	Nutritive value of Olsztyn University students’ diet	30 students; female and male; ages: 19–25	Median Se intake from the diet, 20 μg; average, 25 μg; standard deviation (SD), 0.00175
Spain [26]	Food intake using 1-year food frequency questionnaire (FFQ) and serum Se concentration in elderly people	205 institutionalized elderly women (*n* = 125) and men (*n* = 125)	Se intake μg/day: women, 94.4 ± 23.6; men, 107.1 ± 32.2; animal, but not vegetable protein, contributed to serum Se
Spain [27]	Cross-sectional analysis using a 3-day food record	483 children (216 boys and 267 girls) aged between 8 and 13 years.	91.0 μg/day (above the recommended level in 99.4% of subjects); the main dietary sources were cereals, meats, fish and milk products
Spain [28,29]	Cross-sectional analysis using a 24-h dietary food recall	20 nonagenarian subjects of 90–102 years (92.5 ± 3.5 years; 80% women) and sixty-three 80–90-year-olds (84.0 ± 2.7 years; 58.7% women); all non-institutionalised	Nonagenarians had higher Se intake than the 80–90-year-old group. Se intake was equal to (in the 80–90-year-old group) or slightly above (in the nonagenarians) the recommended intake and was negatively associated with anxiety/depression
Spain [30]	Cross-sectional analysis using 24-h diet recall questionnaire	280 healthy women grouped into three major groups: women aged ≤35 years; women aged 36–45, and finally, women aged >45 years.	74.66 μg/day in 18–35-year-olds; 50.71 μg/day in 36–45-year-olds; 20.56 μg/day in >45-year-olds. There was a significant and positive association between bone mineral density and dietary intake of Se
France [31]	Case-control study of Se in people exposed to Se concentration in drinking water greater than the maximum recommended limit (10 μg/L) using an FFQ	40 exposed subjects and 40 non-exposed controls	Exposed subjects intake 64 ± 14 μg/day; non-exposed 52 ± 14 μg/day
Belgium [32]	To determine the Se status of the population	Intake calculated from analysis of 800 food products combined with food intake records	The mean dietary Se intake was calculated to be 60 μg/day; the major sources of Se intake were meat and meat products (31%), fish and shellfish (20%), pasta and rice (12%) and bread and breakfast cereals (11%)
Republic of Slovenia [33]	Cross-sectional to assess Se status during 3 months of basic military training in a group of recruits using analysis of diet samples	15 recruits	48 ± 10 μg/day
Italy [34]	Cross-sectional study of Se concentration in human milk after delivery compared to infant intake of Se from breast milk	242 women and their breast feeding infants	Mean Se concentration in milk samples was 12.1 ± 3.0 ng/g (lower than that noted in other international studies); mean Se intake in infants was 9.5 ± 2.4 μg/day
Italy [35]	A cross-sectional analysis using a 3-day diet diary	128 haemodialysis patients	28.3 ± 18.1 μg/day
UK [36]	Headline results from Year 1 of the Rolling Programme (2008/2009)	Various age groups	Roughly 50% of adult women and older girls and 20% of men and older boys had Se intakes below the LRNI level
UK [37]	Longitudinal study of healthy British adults using biochemical and molecular biomarkers	Women (*n* = 24, mean age 45 years), males (*n* = 39, mean age 45 years)	Women had a baseline Se intake of 43 μg/day; males had a baseline Se intake of 54 μg/day. The average intake of both sexes was 1.13 μmol/L (89.22 μg/L)
Northern Ireland [38]	Case-control study of chronic heart failure patients using a 4 day food diary	39 subjects and 27 healthy controls	73% of subjects were consuming less than the recommended amount of Se
UK [39]	Cross-sectional analysis using a self-reported 12-month dietary intake questionnaire	1,798 subjects evaluated for prostate cancer risk	A 25% increase in Se was associated with higher insulin-like growth factor I (IGF-I), elevating prostate cancer risk
The Netherlands [40]	To assess the contribution of dairy products to the intake in several life stages	Data derived from 3 Dutch food consumption surveys and the Leiden Longevity Study	In young children, dairy products contributed 21% to the Se intake; in adults and the elderly, the contribution of dairy products to intake was 18%–19%
Northern Italy [41]	Prospective analysis of data from the Hormones and Diet and the Etiology of Breast Cancer (ORDET) cohort using a semi-quantitative FFQ	7182 subjects	55.7 μg/day
Spain [42]	A cross-sectional study analysis using a 3-day food diary	573 Madrid schoolchildren aged 8–13 years	Se intake was also lower in obese children (1.99 ± 0.62 μg/kg) compared to normal weight children (2.73 ± 0.88 μg/kg)
Italy [43]	Semi-quantitative FFQ data from a population-based case-control study to determine the relationship between Se exposure and risk of cutaneous melanoma	54 melanoma patients and 56 controls	Median Se intake was 54 μg/day in the cases and 57 μg/day in the controls

**Table 2:** Studies investigating Se intake and Se concentration in water and food in Middle Eastern countries. KSA, Kingdom of Saudi Arabia.

Country	Study	Subject No./Age/Samples Details	Se Intake/Water and Food Content
Turkey [44]	Se Content of human breast milk in lactating women; dairy milk products of Turkey	Breast milk samples from 10 healthy lactating women collected during 2 months; colostrum milk 7 days and transitional milk 8–15 days postpartum; mature milk on the 15th day postpartum; cow milk samples collected from cities in Turkey from 1994 to 1996; other milk samples collected from various cities in Turkey	Average Se concentration (mean ± SD) in human breast milk: colostrum 13.8 ± 4.71 ng/g; transitional 11.52 ± 1.92 ng/g; mature 11.16 ± 6.15 ng/g; cow milk ranged from 5.78 to 32.02 ng/g, lowest in Van and highest in Aksaray; the overall mean in cow milk was 15.24 ± 5.54 ng/g; other milk: goat milk 39.28 ± 30.11 ng/g; sheep milk 32.28 ± 17.98 ng/g; water buffalo 32.89 ± 15.32 ng/g; goat milk contained almost twice that in cow’s milk
Turkey [45]	Systematic review of trace elements in growth	Summary of various studies investigating Se levels in children	Estimated daily intakes: 30–40 μg/day
Turkey [46]	Trace metal levels in milk and dairy products consumed in middle Anatola, Turkey	Samples collected reflected the potential consumption by the Turkish population.	Highest average Se found in Tulum cheese (0.434 mg/kg); butter averaged 0.315 mg/kg, followed by Kaşar cheese, with 0.276 mg/kg and milk with 0.232 mg/kg; white cheese and drained yoghurt contained 0.159 mg/kg and 0.082 mg/kg, respectively; ice cream, milk and whey powders, yogurt, Ayran or Lor cheese contained no Se.
Jordan [47]	Survey of ground water in semi-arid areas: Amman Zarqa Basin	Se concentration (in μg/L) in the different aquifers in the Amman Zarqa Basin	Total Se content varied markedly in the aquifers; the Se concentration ranged from 2 to 441 μg/L, and the average was 30.8 μg/L
KSA [48]	The distribution of Se in dairy farms: a preliminary report from Al-Kharj	The concentration of Se in wheat grain grown in KSA	Se ranged from 8 to 293 μg/kg (average 78.4 μg/kg); the lowest average, 50.6 μg/kg, was the Wadi Al-Dowasir area, while the highest average, 285.5 μg/kg, was in Al-Jouf
KSA [49]	Survey of foods from the KSA market and estimation of the daily intake	Foods for residents of Jeddah	Intake of 75–121.65 μg Se/person per day
KSA [50,51]	Survey of infant milk formula	Infant formulas	Contained adequate Se for infants up to 6 months
KSA [51,52]	Survey of breast milk and cow’s milk	Breast milk	Breast-fed infants had Se intakes between 0.9 and 15 μg/day
Jordan [53]	Case-control study of colorectal cancer patients using a semi-quantitative FFQ	220 patients and 220 age- and gender-matched controls	Colorectal cancer patients had significantly lower dietary intake of Se than controls (38.75 ± 11.42 μg/day *versus* 59.26 ± 8.91 μg/day)
Tehran, Iran [54]	Cross-sectional analysis of 24-h food recalls	100 female university students aged 18-25 years	Se intake was 54.5 ± 38.7 μg/day
Shahin Shahr and Meymeh, Iran [55]	Cross-sectional analysis of 24-h food recalls	Seventy one 6–7-year-old normal weight children	Se intake was within recommendations
Iran [56]	Case-control analysis of 24-h dietary recall data	445 coronary artery disease patients divided into those with significant disease (>50% occlusion) (Angio+ (*n* = 273)) and those with <50% coronary artery occlusion (Angio− (*n* = 172)) and 443 healthy controls	Se intake in the control 34.98 ± 22.93 mg/day, Angio—48.70 ± 23.53 mg/day and Angio+ 49.63 ± 29.75 mg/day *
Iran [57]	Case-control analysis of a 24-h validated FFQ	47 cases with oesophageal squamous cell carcinoma patients and 96 controls	The calorie adjusted mean Se intakes were categorized into 3 tertiles; controls consumed 623.5-times higher Se than patients
KSA [58]	Quantification of Se in food from local markets of Riyadh.	Primary sources of Se in the diet were: meat and meat products (31%), egg (20.4%), cereals and cereal products (16%), legumes (8.7%), fruits (6.8%), milk and dairy products (2.0%), beverages (2%), sweets (1.8%), pickles (0.2%) and oil (0.02%).	Daily intake of Se was estimated to be 93 μg/day
Iran [59]	Cross-sectional analysis to determine Se intake using a 3-day food record in postmenopausal women	30 postmenopausal women	Daily intake of Se was estimated to be 40 μg/day

* These values are substantially higher than most others provided in Table 2. The corresponding author of the reference was contacted to confirm the units stated in the reference. No response was obtained prior to this manuscript submission. Therefore, this reference was excluded from our interpretation of the data.

A 2008 longitudinal study in healthy British adults reported low Se intake [37]. Subsequently, the National Diet and Nutrition Survey (NDNS) 2008/2009 found that roughly 50% of adult women and older girls and 20% of men and older boys had Se intakes below the lower reference nutrient intake (LRNI) level [36]. A 2009 longitudinal analysis of Se intakes of Caucasian and South Asian women living in the UK who were enrolled in the vitamin D, Food Intake, Nutrition and Exposure to Sunlight in Southern England (D-FINES) study [60] showed that the RNI of Se was not achieved by 80%–90% of Caucasians and by 83%–95% of the South Asians. Likewise, 60% of the former and 60%–70% of the latter failed to meet the LRNI. The number of women that had Se intakes below the LRNI or RNI was not significantly different in terms of ethnicity. Only in the South Asian women was a seasonal variation noted, with significantly lower Se intake in the autumn [20].

#### 3.1.2. Selenium Intake Studies: Middle Eastern Countries

Breast milk analysis in Turkey shows that Se content is below the international reference range set at 18.5 μg/L throughout the lactation period [61]. Analysis of various dairy products in Turkey also indicates that the Se content varies depending on the type of food. Butter and different types of cheeses had quite high concentrations, whereas other dairy products, such as milk and ice cream, contained negligible amounts.

The concentration of Se in water is extremely variable and, in some places, excessive, as reported in parts of Jordan [47]. However, a recent case-control study found Se intakes equivalent to the RDA in control subjects, while patients with colorectal cancers consumed significantly less [53].

Recent studies within Iran have reported adequate Se intakes within the general population [54,55], but extremely low intakes in oesophageal squamous cell carcinoma patients [57].

The concentration of Se in wheat grain grown in the KSA is extremely variable, with one study reporting a range from 8 to 293 μg/kg (median 78.4 μg/kg) [48]. The lowest Se content (average 50.6 μg/kg) was found in wheat from the Eastern parts of KSA, in the Wadi Al-Dowasir area, while the highest levels (average 285.5 μg/kg) was found in the northwest, around the Al-Jouf region. A further investigation of Se in soil, water and alfalfa from Al-Kharj in the centre of the country found that some samples had Se content as low as the low-Se zone in China [48].

Overall, Se intake varied throughout the Middle East. A recent study from Riyadh reported intakes nearly double the RDA [58], while a study from King Abdulaziz University in KSA reported intakes between 75 and 121.65 μg/person per day in a population sample in Jeddah [49]. In this study, the major food groups providing Se were cereals/cereal products, legumes and meats. Another study, including infant formulas, mostly imported from Europe, contained adequate levels of this trace element for infants up to six months [50,51]. However, some breast-fed children may still be at risk of low Se intakes, according to a study by Al Saleh *et al.*, where intakes between 0.9 and 15 μg/day were reported [51,52].

### 3.2. Selenium Status Studies

The literature search located 48 European/UK studies and 44 investigations in the Middle East reporting Se status. A summary of the findings are presented in Table 3 and Table 4.

**Table 3:** Studies investigating Se status in Europe (including the UK).

Country	Study Description	Subject Details/Number	Se Status by Body Compartment
UK [5]	Randomised, double-blind, placebo-controlled trial of Se supplementation on thyroid function	501 males and females aged 60–74 years	Mean baseline plasma Se, 91 μg/L
UK [20,60]	A longitudinal analysis of Se intakes in UK South Asian and Caucasian women: oral presentation	135 Caucasian premenopausal (19–40 years), 135 Caucasian postmenopausal (55–70 years), 39 Asian premenopausal (19–40 years) and 39 Asian postmenopausal (55–70 years) women.	Full plasma GPx expression (serum or plasma Se level of 98.7 μg/L [8] was not achieved by 51.1% of the UK (Caucasian and Asian), 63.3% of UK Asian and 45.2% of UK Caucasian subjects
UK [24]	Retrospective cohort, including previously reported indices of bone health and dietary Se intake and Se analysis (during 2010) of previously collected plasma and serum in the D-FINES study [60].	51 UK resident postmenopausal women (35 Caucasian; 16 Asian, 55–75 years; 41 UK resident premenopausal women (27 Caucasian; 14 Asian), 19–40 years	Mean serum or plasma Se status μg/L in postmenopausal Caucasian and Asian women, respectively, was 100.91 ± 16.94 μg/L and 99.69 ± 23.73 μg/L; in premenopausal Caucasian and Asian women, respectively, it was 96.68 ± 11.76 μg/L and 92.10 ± 17.22 μg/L
Spain [26]	Cross-sectional analysis of food intake and serum Se in elderly people	Dietary intake assessed in 205 institutionalized elderly using 1-year FFQ: women (*n* = 125); men (*n* = 125)	Mean serum Se levels μg/L: women 87.9 ± 16.6; men 86.2 ± 17; bakery, fish and shellfish, meat, meat products and dairy products were associated with serum Se; animal, but not vegetable protein contributed to serum Se
Spain [27]	Cross-sectional analysis using a 3-day food record and serum analysis	483 children (216 boys and 267 girls) aged between 8 and 13 years	The mean serum Se was 71.1 μg/L; however, it was <60 μg/L in 13.9% of subjects, and <45 μg L/ in 5.6%
Republic of Slovenia [33]	A cohort study to assess Se status during 3 months of basic military training	15 recruits	Plasma Se was between 70 and 80 ng/g (** 71.75–82 μg/L; ^##,^ ^^^, #^ 76.87 μg/L)
UK [37]	Longitudinal Se status in healthy British adults: assessment using biochemical and molecular biomarkers	24 women, mean age 45 years; 39 males, mean age 45 years	Women’s baseline plasma Se was 1.11 μmol/L (* 87.65 μg/L). Male’s baseline plasma Se was 1.16 μmol/L (* 91.59 μg/L)
Spain [42]	Cross-sectional study to determine whether being overweight/obesity is associated with children’s Se status	573 Madrid schoolchildren aged 8–13 years	Children with excess weight (BMI > P85) had lower serum Se than those of normal weight (64.6 ± 16.8 μg/L compared to 75.3 ± 12.2 μg/L)
Italy [43]	Population-based case-control series to determine the relationship between Se exposure and risk of cutaneous melanoma	54 melanoma patients and 56 controls	Median plasma Se was 99 μg/L in the cases and 89 μg/L in the controls; median toenail Se was 0.64 μg/g in the cases and 0.65 μg/g in the controls
Greece [62]	Cross-sectional analysis of maternal-neonatal serum Se and copper levels in Greeks and Albanians	Subjects: 1118 Greek and 820 Albanian immigrant mothers	Mean serum Se in Greek mothers was 68.3 ± 8.5 μg/L and Greek new-borns 37.2 ± 8.9 μg/L; mean serum Se in Albanian mothers was 37.4 ± 8.9 μg/L and Albanian new-borns 34.33 ± 9.1 μg/L
Portugal [63]	Cross-sectional analysis of data from healthy Portuguese subjects of the Lisbon population	136 women aged 20–44 years; 47 men aged 45–70 years	Mean serum Se 81 ± 14 μg/L in women; the oldest subjects had the highest concentrations (regardless of gender), although higher serum Se values were noted in men
France [64]	Cross-sectional study: The SUpplementation en VItamines et Mineraux AntioXydants SU.VI.MAX) study	1821 women (30–60 years) and 1307 men (45–60 years)	Baseline mean serum Se for men was 1.13 ± 0.20 μmol/L (* 89.22 ± 15.79 μg/L) and women 1.08 ± 0.19 μmol/L (* 85.28 ± 15.00 μg/L)
Germany [65]	Cross-sectional analysis of Se and antioxidant vitamin status	178 elderly women (absence of severe disease; mean age: 63.2 years (SD ± 2.73)	Mean serum Se in omnivores (*n* = 159) 1.17 ± 0.22 μmol/L (* 92.38 μg/L) and vegetarians 1.09 ± 0.22 μmol/L (* 86.07 μg/L); sixty five (38.9%) women had serum Se concentrations below 1.1 μmol/L (* 86.86 μg/L) and 153 (91.6%) below 1.5 μmol/L (* 118.44 μg/L)
Czech Republic (West Bohemia) [66]	Cross-sectional analysis to assess Se deficiency of West Bohemia population	Age: 6–65 years; sex: male and female	Serum mean Se: 55.4 ± 13.8 μg/L; urine: 15.4 ± 5.7 μg/L; 13.6 ± 6.0 μg/g creatinine. hair: 0.268 ± 0.051 μg/g Se indexes were concluded to be low
EU, various countries [67]	Nested case-control assessing plasma Se and prostate cancer risk: results from the European Prospective Investigation into Cancer and Nutrition (EPIC)	959 men (with incident prostate cancer) 1059 matched controls	Mean plasma Se in controls ranged from 63.2 μg/L to 76.6 μg/L; lowest levels in Greece and Germany and highest in the UK and Sweden; plasma concentrations in EPIC (mean: 70 μg/L)
Estonia [68]	Cross-sectional analysis of 5 studies between 1993 and 2001	404 subjects included women, pregnant women and men aged 19.5 to 52 years, but not declared in all studies	Serum Se concentration ranged from 26 to 116 μg/L (mean: 75 μg/L), which were comparable to analyses that were completed in the Finnish population prior to the use of Se-enriched fertilizers
Denmark [69]	Cross-sectional analysis of serum Se and selenoprotein P status in adult Danes within an 8-year follow up	817 subjects: women, 18–22 years, 40–45 years, 60–65 years; men, 60–65 years.	Mean serum Se 98.7 ± 19.8 μg/L; serum Se increased with age in both sexes and decreased by 5% on average from 1997 to 1998; there was a weak correlation between serum Se and fish intake; Se status was concluded to be acceptable
UK [70]	Cross-sectional analysis of Se blood levels	68 pre-term infants (35 boys and 33 girls)	Mean plasma Se at term and 6 months were 0.49 ± 0.15 μmol/L (* 38.69 μg/L) and 0.72 ± 0.14 μmol/L (* 56.85 μg/L) and red blood cell Se 1.68 ± 0.40 μmol/L (* 132.65 μg/L) and 1.33 ± 0.19 μmol/L (* 105.02 μg/L), respectively
UK [71]	Cross-sectional analysis of Se status and its correlates in a British National Diet and Nutrition Survey (NDNS)	883 independent and 251 institution living men and women, aged 65 years and over	Plasma mean Se 0.94 μmol/L (* 74.22 μg/L) for independents and plasma mean Se 0.90 μmol/L (* 71.06 μg/L) for those in the institutional setting
Germany [72]	Cross-sectional analysis of participants in the Lipid Analytic Cologne cohort	792 participants who never smoked, who did not use antihypertensives and who did not have diabetes or known atherosclerotic disease	Mean serum Se concentration was 68 ± 32 μg/L
5 European Cities [73]	A 6-year prospective study of fracture-related factors: the Osteoporosis and Ultrasound Study (OPUS)	2374 postmenopausal women	Mean baseline serum Se was 94.3 (range 54.4–161.2) μg/L; the mean of the low quintile was 69.50 (range 39.46–77.06) μg/L, and the mean of the high quintile was 128.43 (range 115.99–205.25) μg/L; higher Se was associated with higher hip bone mineral density (BMD) at study entry
The Netherlands [74]	A 16.2-year prospective longitudinal study of The Netherlands cohort	120,852 subjects aged 55–69 years completed a dietary and lifestyle questionnaire on dietary habits and provided toenail clippings for baseline Se determination	Mean Se concentration was approximately 0.563 μg/g at baseline and did not change significantly throughout the study
Denmark [75]	A 16-year longitudinal follow up of the Copenhagen Male Study	3333 males aged 53–74 years	167 (5.1%) subjects died of lung cancer: 5.0% of them with low serum Se (0.4–1.0 μmol/L) (* 31.58–78.96 μg/L), 5.1% with medium serum Se 1.1–1.2 μmol/L (* 86.86–94.75 μg/L) and 5.1% with high serum Se 1.3–3.0 μmol/L (* 102.65–236.88 μg/L)
UK [76]	Baseline data from a double-blind, placebo-controlled, pilot trial	230 primiparous pregnant women	The overall median whole-blood Se concentration at baseline was 1.31 (* 103.44 μg/L) (range 0.84–3.33) μmol/L (* 66.33–262.94 μg/L)
UK [77]	Cross-sectional analysis of anthropometric indices with Se-status biomarkers using data from 2000 to 2001 (NDNS)	1045 (577 female, 468 male) British Caucasian adults ages 19–64	Median plasma and erythrocyte Se concentrations were 1.08 (0.98, 1.20) μmol/L (* 85.28 (77.38, 94.75) μg/L) and 1.62 (1.38, 1.91) μmol/L (*127.92 (108.96, 150.81) μg/L), respectively; for males, values were 1.09 (0.99, 1.22) μmol/L (* 86.07 (78.17, 96.33) μg/L) and 1.54 (1.34, 1.79) μmol/L (* 121.60 (105.81, 141.34) μg/L), respectively; for females 1.07 (0.97, 1.18) μmol/L (* 84.49 (76.59, 93.17) μg/L) and 1.71 (1.43, 1.99) μmol/L (* 135.02 (112.91, 157.13) μg/L), respectively
UK [78]	Data from the UK PRECISE (Prevention of Cancer by Intervention with Selenium) pilot, randomized, double-blind, placebo-controlled trial	501 elderly volunteers	Mean plasma Se concentration was 88.5 ng/g (** 90.71 μg/L) at baseline
UK [79]	Data from the about Teenage Eating Study, a prospective observational study	126 pregnant adolescents (14–18-year-olds) between 28 and 32 weeks gestation	Plasma Se (mean ± (SD)) was lower in the small-for-gestational-age group (*n* = 19: 49.4 ± 7.3 μg/L) compared with the appropriate-for-gestational-age group (*n* = 107: 65.1 ± 12.5 μg/L); smokers had lower Se compared with non-smokers (*p* = 0.01), and Afro-Caribbean women had higher Se compared with white Europeans (*p* = 0.02)
UK [80]	Randomised, double-blind, placebo-controlled trial	119 free-living, non-smoking men and women, aged 50–64 years	Mean baseline plasma Se was approximately 90 ng/mL = 90 μg/L
UK [81]	Cross-sectional analysis of Se status with cardio-metabolic risk factors in the general population	1042 white participants (aged 19–64 year) from the 2000–2001 UK NDNS	Mean plasma Se concentration was 1.10 ± 0.19 μmol/L (* 86.86 ± 15 μg/L)
UK [82]	Randomized, placebo-controlled, parallel-group study stratified by age and sex	501 volunteers aged 60 to 74 years	Mean plasma Se was 88.8 ng/g (** 91.02μg/L) at baseline
Finland [83]	Cross-sectional and longitudinal associations of serum Se with cardiovascular risk factors	1235 young Finns aged 3–18 years and followed from 1980 until 1986	Mean (±SD) serum Se was 74.3 ± 14.0 ng/mL = 74.3 ± 14.0 μg/L at baseline and 106.6 ± 12.5 ng/mL = 106.6 ± 12.5 μg/L six years later
UK, Belgium, Italy [84]	Cross-sectional analysis of data from the Dietary Habit Profile in European Communities with Different Risk of Myocardial Infarction: the Impact of Migration as a Model of Gene-Environment Interaction (IMMIDIET) study to evaluate the effect of genetic and dietary habits on cardiovascular disease risk factors in non-diabetic subjects	Couples randomly recruited from general practice in southeast London, England (*n* = 263), the Flemish territory of Belgium (*n* = 267) and the Abruzzo region of Italy (*n* = 271)	The mean (SD) plasma Se (in μmol/L) in men without metabolic syndrome was 1.24 (* 97.91 μg/L) (0.23) and with metabolic syndrome was 1.24 (* 97.91 μg/L) (0.23); similar results for women without metabolic syndrome were 1.21 (* 95.54 μg/L) (0.23) and with metabolic syndrome 1.32 (* 104.23 μg/L) (0.28)
Poland [85]	Case-control to estimate Se concentration, glutathione peroxidase activity and total antioxidant status in patients with multiple sclerosis and the influence of dietary habits	101 patients with relapsing-remitting MS (aged 18–58 years) and 63 healthy people (aged 19–65 years)	Serum Se in patients was 55.2 ± 16.2 μg/L compared with controls 79.2 ± 20.6 μg/L
Poland [86]	Cross-sectional study to determine serum and urinary Se in children with and without obesity	80 children (age 6–17; 40 boys, 40 girls)	Serum Se (in μg/L) in control girls 102.3 ± 7.9, control boys 111.1 ± 9.5, obese girls 80.4 ± 8.2, obese boys 82.8 ± 10.3; urine Se (in μg/L) in control girls 55.9 ± 9.4 in control boys 60.3 ± 11.5, in obese girls 36.0 ± 7.5, in obese boys 36.7 ± 5.6
Poland [87]	Case-control analysis of anti-oxidant defences in pubertal patients with type 1 diabetes mellitus (T1DM) and their siblings	87 children with T1DM, 2–19 years old and their siblings comprised of 27 and 41 children, aged 4.5–16.5 years and 10.5–18 years, respectively, and 41 healthy children aged from 10.5 to 18 years	Plasma mean Se (μg/L) in T1DM children was 58.4, in their sibling was 53.45 and in the controls was 53.3
Poland [88]	Case-control study in Szczecin, a region of northwestern Poland	95 lung cancer cases, 113 laryngeal cancer cases and corresponding healthy controls	Among lung cancer cases, mean serum Se was 63.2 μg/L, compared to 74.6 μg/L for their matched controls. Among laryngeal cancer cases, the mean serum Se was 64.8 μg/L, compared to 77.1 μg/L for their matched controls
Austria [89]	Case-control study of people with autoimmune thyroiditis	Patients with autoimmune thyroiditis and matched group of healthy persons	Serum Se in the patients was 98.0 ± 15.6 μg/L and in controls was 103.2 ± 12.4 μg/L
Finland [90]	Systematic review	Sampling of human blood from the same 60 adults, annually	Mean plasma Se increased from 0.89 μmol/L (* 70.27 μg/L) in the 1970s to a general level of 1.40 μmol/L (* 110.54 μg/L) since introducing Se-supplemented fertilizers in 1984
Germany [91]	Cross-sectional study in disorders in wound healing	44 trauma patients with disorders in wound healing	Mean plasma Se was 0.79 ± 0.19 μmol/L (* 62.38 ± 15.00 μg/L)
Germany [92]	Prospective observational study to assess the effects of perioperative sodium-selenite administration on Se blood concentrations in cardiac surgical patients	104 cardiac surgical patients	Whole blood Se was 89.05 ± 12.65 μg/L pre-surgery and 70.84 ± 10.46 μg/L post-surgery
Germany [93]	Prospective observational study in the development of multi-organ dysfunction in cardiac surgical patients	60 patients (age 65 ± 14 years) undergoing cardiac surgery with the use of cardiopulmonary bypass	Fifty patients exhibited a significant Se deficiency already before surgery. Se was significantly reduced after the end of surgery when compared to preoperative values (89.05 ± 12.65 to 70.84 ± 10.46 μg/L)
Greece [94]	A prospective cross-sectional study to assess the relationship of urine Se levels with thyroid function and autoimmunity in pregnant women	47 euthyroid women in uncomplicated singleton pregnancies (mean age + SD: 30 + 5 years)	Urine Se in μg/L: 1st trimester 91 ± 60, 2nd trimester 82 ± 59, 3rd trimester 69 ± 57
Denmark [95]	Case-control study in patients with newly-diagnosed autoimmune thyroid disease	97 patients with newly-diagnosed Graves’ disease, 96 with hypothyroidism, 92 euthyroid subjects with high serum thyroid peroxidase antibody and 830 controls	Mean serum Se in Graves’ disease was 89.9 μg/L, in euthymic subjects 98.4 μg/L and in controls 98.8 μg/L
Spain [96]	Cross-sectional study of a healthy population	84 healthy adults (31 males and 53 females) from the province of Granada	Mean plasma Se was 76.6 ± 17.3 μg/L (87.3 ± 17.4 μg/L in males, 67.3 ± 10.7 μg/L in females), whereas the mean erythrocyte Se was 104.6 μg/L (107.9 ± 26.1 μg/L in males and 101.7 ± 21.7 μg/L in females)
Spain [97]	Cross-sectional study of a healthy population in southern Spain	340 subjects	86.5% had plasma Se below 125 μg/L
Hungary [98]	A single-centre cross-sectional clinical survey following cardiac surgery	197 consecutive patients undergoing on-pump operation	Mean blood Se was significantly lower in non-survivors 102.2 ± 19.5 μg/L compared with survivors 111.1 ± 16.9 μg/L
The Netherlands [99]	Prospective cross-sectional analysis during early gestation to determine the impact of Se status on the risk of preterm births	1197 white Dutch women with a singleton pregnancy followed from 12 weeks’ gestation	Serum Se at 12 weeks’ gestation was significantly lower among women who had a preterm birth than among those who delivered at term mean 0.96 ± 0.14 μmol/L *vs*. 1.02 ± 0.13 μmol/L (* 75.80 ± 11.05 μg/L *vs*. * 80.54 ± 10.26 μg/L)
France [100]	The prospective, longitudinal Epidemiology of Vascular Ageing (EVA) study	1389 subjects aged 59–71 years followed for 9 years	Mean baseline plasma Se was 1.08 ± 0.21 μmol/L (* 85.28 ± 16.58 μg/L) in men and 1.10 ± 0.20 μmol/L (* 86.86 ± 15.79 μg/L) in women
Italy, Slovenia, Croatia, Greece [101]	Prospective cohort with four recruitments areas to assess Se exposure due to fish consumption in the Mediterranean area	Various tissue samples from 900 Italian, 584 Slovenian, 234 Croatian and 484 Greek women	Mean Se concentrations: Italian mother’s blood 117 ng/g, Italian cord blood 113 ng/g Italian breast milk 18 ng/g; Slovenian cord blood 76 ng/g, Slovenian breast milk 17 ng/g; Croatian mother’s blood 90 ng/g, Croatian cord blood 96 ng/g, Croatian breast milk 18 ng/g; Greek cord blood 104 ng/g, Greek breast milk 21 ng/g; Italian urine 31 μg/g; Croatian urine 4 μg/g, Greek urine 24 μg/g

* Se μg/L calculated using an atomic weight of 78.96; ** converted from ng/g using the density of plasma at approximately 1.025 g/mL; ^#^ not clear if it is a mean value; ^^ calculated average; ^##^ sex not established.

**Table 4:** Studies investigating Se status in Middle Eastern countries.

Country	Study Description	Subject Details/Number	Se Status by Body Compartment
KSA [21,102]	Cross-sectional analysis of Serum and toenail Se in the Al-Kharj district	Males and females 743 serum and 691 toenail samples	Mean serum Se: 107.045 ± 23.045 μg/L and toenail Se 0.634 ± 0.221 μg/g
Kuwait [22]	Cross-sectional analysis of Se and its chemical forms in the milk of Kuwaiti and Non-Kuwaiti lactating mothers	34 donors (age 25 to 40 years) Kuwaitis *n* = 17, non-Kuwaitis *n* = 17	Mean ± SD of milk Se (μg/L) 0–6-month lactation period were 20 ± 0.8 and 16 ± 0.4 in Kuwaiti and non-Kuwaiti women, respectively; mean ± SD of milk Se (μg/L) 6–12-month lactation period were 18 ± 0.4 and 15 ± 0.2 in Kuwaiti and non-Kuwaiti women, respectively; in both Kuwaiti and non-Kuwaiti women, the milk Se was significantly lower at the 6–12-month lactation period compared to the 0-6-month one (*p* < 0.05*)*; in addition, Kuwaiti women had significantly higher milk Se levels during both the 0–6-month and 0–12-month lactation periods
Turkey [23]	Cross-sectional analysis of Se in mothers and their neonates using hair, breast milk, meconium and maternal and umbilical cord blood	29 pairs of mothers and their new-born babies	Average Se concentrations in blood plasma: 68.5 ± 3.6 ng/g (~70 μg/L using density of blood plasma 1.025 g/mL)
Turkey [45]	Systematic review of trace elements in growth: iodine and Se status of Turkish children	Summary of a number of studies investigating Se status in children	1989 study: 76 children aged 2 months–13 years had serum Se of 88 ± 12 μg/L; 1994 study: mean cord blood Se 45 ± 10 μg/L, 2–12 month infants 69 ± 13 μg/L, children >12 months and up to age 16 years 77 ± 12 μg/L; 1996 study: serum Se in boys of 75 ± 9 μg/L and in girls 65 ± 10 μg/L; 2001 study involving 250 children from 4 regions found average serum Se of 51–58 μg/L; a 2004 study of 43 healthy children had a mean serum Se of 74 ± 11 μg/L; a 2002 study in school children from an endemic goitre area 31 ± 23 μg/L; taking into account all of the available data, the estimated range for serum Se was ~50–70 μg/L
KSA [58]	Cross-sectional analysis of serum Se in randomly-selected, healthy, non-smoking adults	140 men and 120 women	Mean serum Se was 100 ± 30.5 μg/L
Turkey [103]	Cross-sectional analysis of serum Se in healthy residents of different ages in Ankara	218 healthy individuals at different ages; cord blood at birth; 2-month infants, 12-month infants; children from >12 months to 16 years; adults 18 to 48 years	Mean Se in cord blood at birth: 45 ± 10 μg/L; mean serum Se 2 months and 12 months: 69 ± 13 μg/L; children >12 to 16 years: 77 ± 12 μg/L; adults 18 to 48 years: 74 ± 16 μg/L
Turkey [104]	Cross-sectional, prospective study to evaluate serum Se in women with gestational diabetes mellitus, glucose intolerants and normal controls	178 pregnant women undergoing 50-g oral glucose tolerance test (OGTT) between 24 and 28 weeks of gestation; age range 24–25 years	Group A (control, *n* = 101) mean serum Se 50 ± 9.8 μg/L; Group B (abnormal 50-g and abnormal 100-g OGTT, *n* = 30) mean serum Se 34.7 ± 8.7 μg/L; Group C (glucose intolerant (GIT), *n* = 49) mean serum Se level 39.9 ± 6.5 μg/L
Iran [105]	Cross-sectional analysis of serum Se in healthy individuals living in Tehran	184 healthy inhabitants, male and female; 24 women (over 16 years)	Mean ± SD serum Se level was 93.9 ± 13.6 μg/L for women and 102.2 ± 12 μg/L for men. The intake in men was statistically significantly higher than in women *p* < 0.0005
Iran [106]	Cross-sectional analysis of serum Se concentration in healthy children living in Tehran	Serum samples of 216 healthy children were analysed; age 0–16 years	Mean ± SD of serum Se (μg/L); <4 years (*n* = 67), male and female, 63.72 ± 20.29; ≥4 years (*n* = 149), male and female, 75.82 ± 13.42
Iran [107]	Cross-sectional analysis of serum Se as a possible factor for persistent goitre in Iranian school children	Schoolchildren were assessed for serum Se concentration as part of an assessment for thyroid health; *n* = 1188 total; serum Se assessment *n* = 500; male and female	Mean ± SD of serum Se (μg/L); overall 119.1 ± 31; boys 108.4 ± 26.2; girls 127.7 ± 32.1; the difference between the sexes was statistically significant
Iran [108]	Cross-sectional analysis of serum Se in healthy volunteers living in Tehran	60 females and 55 males living in Tehran; age 6–62	Mean serum Se was 99.10 ± 21.78 μg/L; there was a significant difference between males and females (males 104.31 ± 24.70 μg/L and females 94.33 ± 17.6 μg/L (*p* < 0.05)
Iran [109]	Cross-sectional analysis of serum Se and glutathione peroxidase concentrations in Iranian patients with angiography-defined coronary artery disease (CAD)	99 males and 53 females with CAD; 31 males and 30 females with normal angiogram; 37 male and 33 female healthy controls	Mean serum Se for the 3 groups was 1.118 ± 0.394 μmol/L (* 88.28 ± 31.11 μg/L), 1.123 ± 0.360 μmol/L (* 88.67 ± 28.43 μg/L) and 1.042 ± 0.364 μmol/L (* 82.28 ± 28.74 μg/L), respectively; serum Se was not significantly different between patients with or without CAD and the control group
Iran [110]	Cross-sectional analysis of Se in healthy women in Tabriz, Iran	60 women of child bearing age and 60 post-menopausal women	Women of child-bearing age mean serum Se 77.72 ± 16.96 μg/L; post-menopausal mean serum Se 72.60 ± 19.08 μg/L; average serum Se was 76.67 ± 17.98 μg/L; no significant difference between age groups; 57.5% had serum Se concentrations under 80 μg/L
Iran [111]	Randomised, double-blind placebo-controlled trial in premature (pre-labour) rupture of membranes	166 primigravid women within age range 16–35 years	Active participants: Serum Se 122.5 ± 23.2 μg/L; controls: 122.9 ± 26.9 μg/L; no significant difference between groups
Lebanon [112]	Cross-sectional analysis of Se and correlates with metabolic syndrome components	159 men and 284 women; age 18–65 years from health centres across Lebanon	Mean ± SD plasma levels were: men 151.2 ± 25.7 μg/L; women 135.0 ± 24.4 μg/L; combined 141.5 ± 26.1 μg/L
Yemen [113]	Cross-sectional analysis of Se status in respiratory syncytial virus (RSV) and human metapneumovirus (HM)	136 males and 36 females with RSV, median age 2 months; 28 male and 11 female children with HM, median age 7 months; 18 male and 6 female that were co-infected, median age 3 months	Se concentrations were low regardless of the virus identified, did not vary with C-reactive protein, and there was no association between the viruses and Se status
Jordan [114]	Cross-sectional analysis of Se in blood	Subjects: 73 total; 56 smokers; 17 non-smokers	Mean blood Se in smokers ranged from 0.269 mg/L (269 μg/L) to 0.368 mg/L (368 μg/L), with an average of 0.332 mg/L (332 μg/L); non-smokers mean blood Se ranged from 0.154 mg/L (154 μg/L) to 0.216 mg/L (216 μg/L), with an average of 0.187 mg/L (187 μg/L)
KSA [115]	Cross-sectional analysis of Se in blood and serum	New-borns of 300 women living in the Al-Kharj district	Mean umbilical cord blood Se levels were 40.8 ± 8.96 μg/L; serum Se in pre-term 32 ± 8.029 μg/L and full-term infants 41.323 ± 8.784 μg/L
KSA [116]	Cross-sectional analysis of Se in serum and toenails in the Al-Kharj district	513 children	Only 1.4% of children had serum Se levels below 45 μg/L; however, 53.4% of the tested children had toenail Se below 56 μg/g
KSA [117]	Case-control study	11 control obese >30 BMI and gestational diabetic obese >30 BMI	Serum Se concentrations for the obese control and diabetic groups were 89 μg/L and 85.1 μg/L, respectively
KSA [118]	Cross-sectional analysis to determine the relationship between Se, thyroid function and other coronary risk factors	140 subjects without overt coronary heart disease stratified by age	Mean serum Se (μmol/L) was 0.4 in <30-year-olds (* 31.58 μg/L), 0.48 in 31–48 year olds (* 37.90 μg/L), 0.51 in ≥49 year olds (* 40.27 μg/L); mean urine Se (μmol/mol creatinine) was 1.48 in <30-year-olds, 1.29 in 31–48-year-olds, 1.07 in ≥49-year-olds
KSA [119]	Case-control study	170 diabetics (19 type I and 151 type 2) with an equal number of controls	Mean (Standard error of mean (SEM)) urinary Se concentration was 0.394 (0.017) μM or (31.1 (1.3) μg/L) and mean (SEM) urinary Se/mol creatine concentration was 56 (2.9) μM or (39.1 (2) μg/g creatinine)
KSA [24]	Retrospective cohort, including previously reported indices of bone health and dietary Se intake and Se analysis (during 2010) of previously collected plasma and serum in Jeddah;Saudi Arabian Bone Health Study (SABHS) study [120].	42 Saudi postmenopausal (45–60 years) and 34 Saudi premenopausal (20–30 years) women living in Jeddah, KSA	Mean plasma or serum levels for postmenopausal 91.24 ± 15.54 μg/L and premenopausal 86.63 ± 10.52 μg/L
Iran [121]	Randomized, double-blind, placebo-controlled trial to examine the effect of prenatal Se on postpartum depression	166 primigravid pregnant women in the first trimester of pregnancy	Baseline mean serum Se in the treatment group was 122.5 ± 23.2 μg/dL (1,225 ± 232 μg/L) and in the control group was 122.9 ± 26.9 μg/dL (1229 ± 269 μg/L) **
Iran [122]	Case-control study to determine the relationship between serum Se and simple febrile seizures in children	30 children with simple febrile seizures and 30 controls (febrile children without seizure)	Mean serum Se in the case and control groups was 44.4 ± 10.9 (444 ± 109 μg/L) and 63 ± 9.78 μg/dL (630 ± 97.8 μg/L), respectively **
Iran [123]	Case-control study to evaluate the correlation of serum Se in patients with Behcet’s disease	46 Behcet’s disease patients and 46 healthy controls	Mean serum Se of patients was 66.4 ± 15.38 μg/L, significantly lower than in the healthy controls (86.87 ± 17.18 μg/L)
Iran [124]	Cross-sectional analysis to determine the serum Se among prisoners in the central jail of Mashhad, northeast Iran	435 prisoners (387 men (34.5 ± 10 years) and 48 women (36.4 ± 11 years))	Mean serum Se was 121 ± 20 μg/L; the prevalence of Se deficiency was 9.7% in the selected sample and was more prevalent in women than men
Iran [125]	Case-control study to determine serum Se in congestive heart failure	77 patients (68.4 ± 10.4 years old; 40.3% female) and 73 healthy volunteers (64.9 ± 4.7 years old; 35.6% female)	Mean serum Se patients 185.9 ± 781.2 μg/L; mean serum Se controls 123.3 ± 115.5 μg/L
Iran [126]	Case-control study to determine serum Se in pemphigus vulgaris patients	43 newly-diagnosed pemphigus vulgaris patients and 58 healthy controls	Mean serum Se in patients 80.34 ± 1.55 μg/dL (803.40 ± 15.50 μg/L); in controls 92.85 ± 1.73 μg/dL (928.50 ± 17.30 μg/L) **
Iran [127]	Case-control study to compare serum Se status in women with or without pre-eclampsia	60 pre-eclampsia patients and 60 healthy subjects	Mean serum Se was 8.82 ± 2.10 μg/dL (88.20 ± 21.00 μg/L) in patients and 10.47 ± 2.78 μg/dL (104.70±27.80 μg/L) in controls
Iran [128]	Case-control study to determine serum Se in different forms of leishmaniasis	95 patients with cutaneous leishmaniasis, 60 with visceral leishmaniasis and 100 controls	Mean serum Se was 3.65 ± 0.88 μg/dL (36.50 ± 8.80 μg/L) in leishmaniasis patients and 11.10 ± 2.37 μg/dL (111.00 ± 23.70 μg/L) in controls
Iran [129]	A nested case-control study to determine changes in plasma Se in women with preeclampsia compared to those with normal pregnancy	38 women with preeclampsia and 38 women having a normal pregnancy	Mean plasma Se was 70.63 ± 21.41 μg/L in preeclampsia women and 82.03 ± 15.54 μg/L in women with normal pregnancy
Iran [130]	Case-control study to determine plasma Se in women with preeclampsia compared to those with normal pregnancy	40 preeclamptic and 40 healthy pregnant women in 34–39th week of gestation	Mean plasma Se in preeclampsia women was 51.75 ± 11.62 μg/L and in controls was 58.51 ± 11.85 μg/L
Turkey [131]	Case-control study to evaluate the possible associations between serum Se and idiopathic intractable epilepsy	70 idiopathic intractable epilepsy patients and 60 healthy matched children	Mean serum Se was 4.40 ± 1.75 μmol/L (* 347.42 μg/L ± 138.18) in epileptic and 5.77 ± 1.88 (* 455.60 ± 148.44 μg/L) in controls μmol/L **
Turkey [132]	Case-control study to evaluate the effects of pica and iron-deficiency anaemia on oxidative stress and antioxidant capacity	47 children with iron-deficiency anaemia plus pica, 22 children with iron-deficiency anaemia only and 21 non-anaemic children as controls	Mean serum Se was 57.5 + 11.2 μg/L in those with pica, 62 + 11 μg/L in those with iron-deficient anaemia only and 80.9 + 13.1 μg/L in controls
Turkey [133]	Case-control study to evaluate serum Se in relation with hyperandrogenism and insulin resistance in women with polycystic ovary syndrome (PCOS)	36 cases with a diagnosis of PCOS and 33 age- and BMI-matched healthy women	Mean serum Se was 41.8 ± 1.8 μg/L in the PCOS women and 49.7 ± 1.6 μg/L in controls
Turkey [134]	Case-control study to investigate the plasma and erythrocyte Se in H1N1-infected children	11 infected children (4 girls, 7 boys; mean age 9.3 ± 4.5 years) and 12 controls (6 girls, 6 boys; mean age 10.8 ± 2.6 years)	Mean plasma Se was 117.54 μg/L in infected children and 132.27 μg/L in controls; erythrocyte Se was 529.71 μg/L in infected children and 599.72 μg/L in controls
Turkey [135]	Case-control study aimed at investigating serum Se in postmenopausal women with osteoporosis and osteopenia	37 osteopenic and 35 osteoporotic women and 35 healthy controls	Mean serum Se was 66.89 ± 15.52 μg/L in osteopenic, 66.16 ± 12.13 μg/L in osteoporotic and 67.12 ± 11.61 μg/L in control subjects
Turkey [136]	A case-control study to evaluate the serum and seminal plasma Se in men with idiopathic infertility	44 patients with idiopathic male infertility and abnormal sperm parameters and 15 subjects with normal sperm parameters and proven fertility	Mean serum Se in μg/L was 138.1 ± 12.3 in normozoospermia, 81.9 ± 36.3 in oligozoospermia; mean semen Se in μg/L was: 81.4 ± 5.4 in normozoospermia, 33 ± 18.2 in oligozoospermia
Egypt [137]	A case-control study to estimate serum sein beta-thalassemia-major patients	108 patients with beta-thalassemia-major and 60 age- and sex-matched healthy children	Mean serum Se was 31.5 ± 19.1 μg/L in cases and 65.9 ± 6.3 μg/L in controls
Egypt [138]	A case-control study to assess Se in obese children and the relationships with serum leptin and metabolic risk factors of obesity	80 obese children (BMI ≥95th percentile for age and gender) and 80 healthy, age- and gender-matched, non-obese controls	Mean serum Se was 63.6 ± 15 μg/L in the obese compared to 78.3 ± 18 μg/L in controls
Egypt [139]	A case-control study to evaluate the oxidant-antioxidant status in children with breath-holding spells	67 children with breath-holding spells (18 pallid and 49 cyanotic) were compared with 60 healthy children	Mean serum Se in μg/dL was 4.01 ± 2.62; in pallid (40.10 ± 26.20 μg/L) 3.98 ± 1.86; in cyanotic (39.80 ± 18.60 μg/L) 8.33 ± 2.16; in controls (83.30 ± 21.60 μg/L)
Yemen [140]	A case-control study to evaluate nutritional factors associated with chronic suppurative otitis media (CSOM)	75 children with CSOM and 74 healthy controls	Mean serum Se was 1.0 μmol/L (* 78.96 μg/L) in cases and 1.2 μmol/L (* 94.75 μg/L) in controls
Kuwait [141]	A case-control study to compare serum Se in morbidly obese female patients seeking bariatric surgery with those of age-matched females with body mass index ≤30.	66 morbidly obese female patients and 44 female controls	Mean serum Se was 86.08 μg/L in the obese group and 101.14 μg/L in the control group

* Se μg/L calculated using an atomic weight of 78.96; ** these values are substantially higher than most others provided in Table 4. The corresponding author of the reference was contacted to confirm the units stated in the reference. No response was obtained prior to this manuscript submission. Therefore, this reference was excluded from our interpretation of the data.

**Figure 2:**
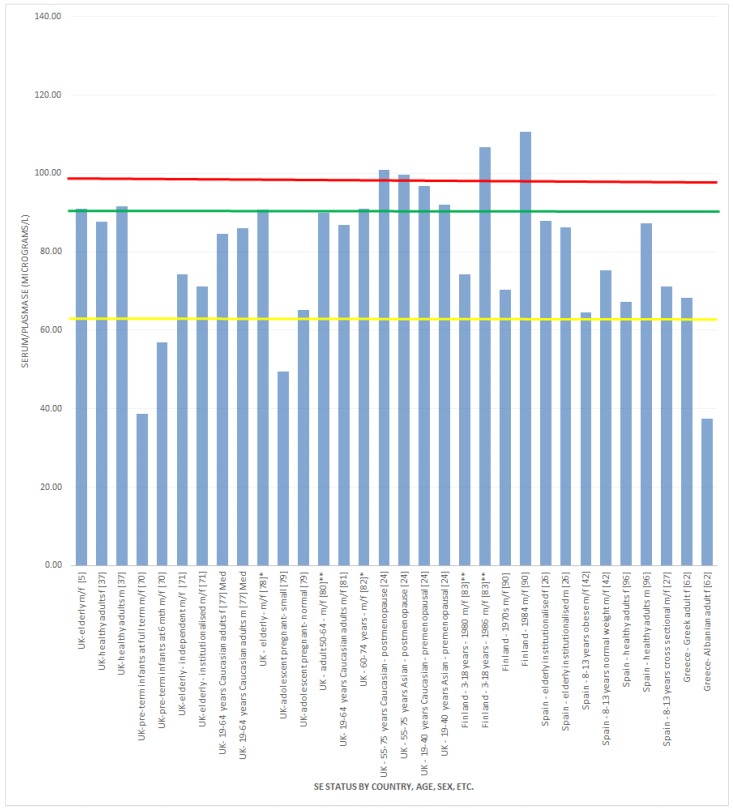

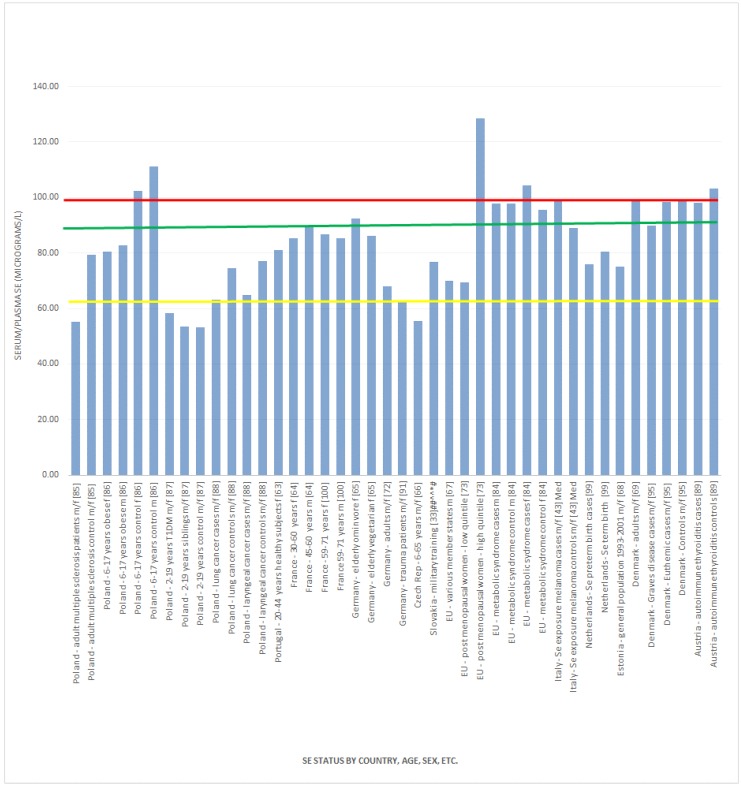
Se status (European + UK regions) *versus* Se concentration required to optimise plasma GPx activity. f = female; m = male; Med = median value; ^#^ not clear if it is a mean value; ^^ calculated average; ^##^ sex not established; * converted from ng/g (plasma density 1.025g/mL); ** converted from ng/mL. The red line 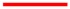, indicates concentration required to optimise plasma GPx activity according to Alfthan *et al.* (98.7 μg/L) [8]. The green line 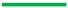, indicates concentration required to optimise plasma GPx activity according to Duffield *et al.* (90.01 μg/L) [7]. The yellow line 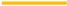, indicates concentration required to optimise plasma GPx activity according to the WHO (63.16 μg/L) [61]

**Figure 3:**
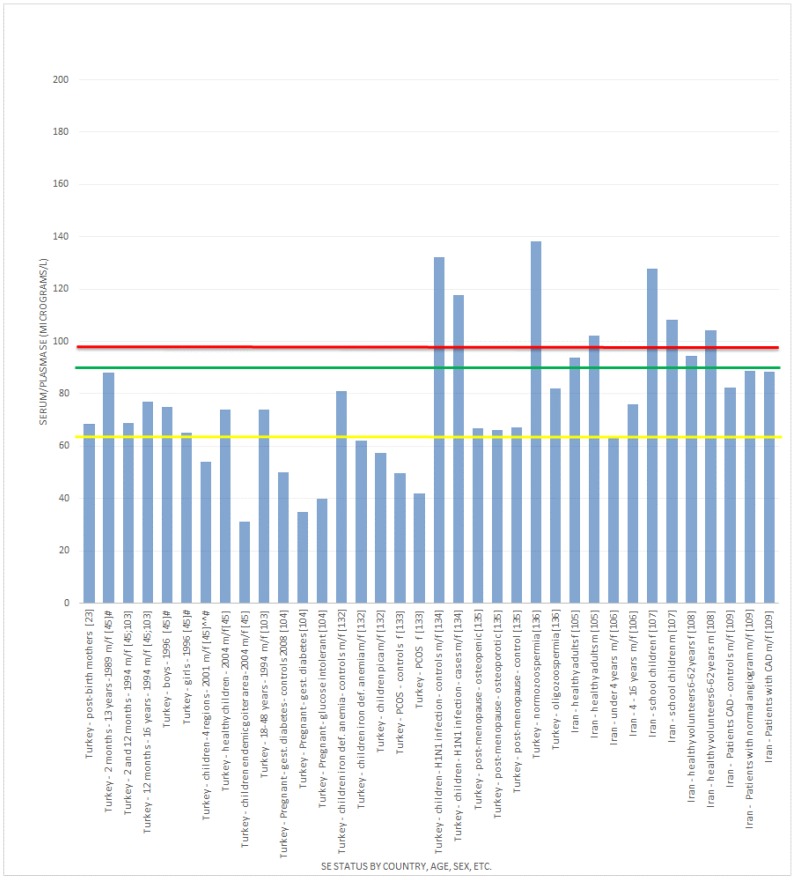

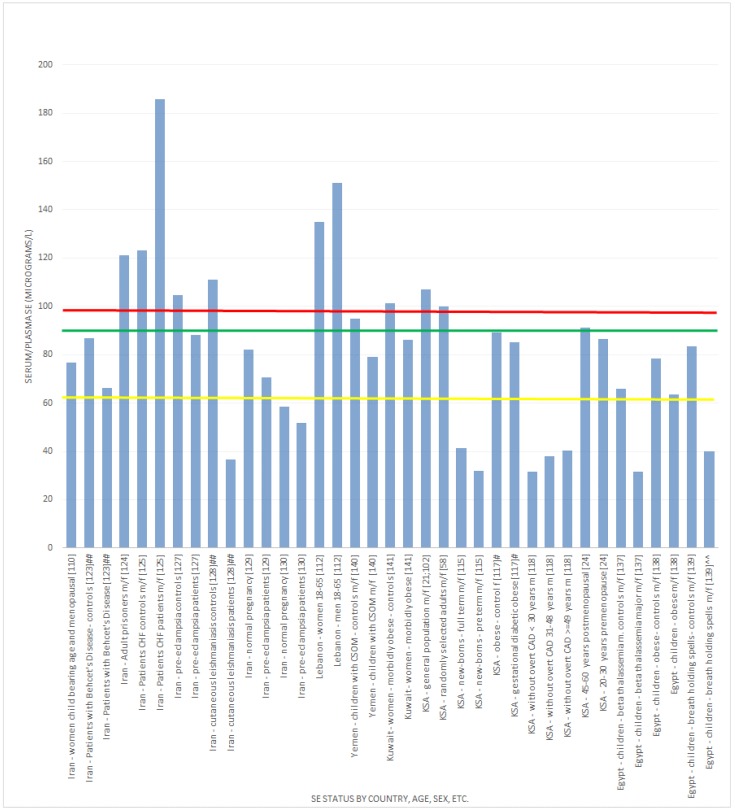
Se status (Middle East regions) *versus* Se concentration required to optimise plasma GPx activity. ^^ calculated average; ^#^ not clear if it is a mean value; f = female; m = male; ^##^ sex not established. The red line 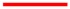, indicates concentration required to optimise plasma GPx activity according to Alfthan *et al.* (98.7 μg/L) [8]. The green line 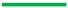, indicates concentration required to optimise plasma GPx activity according to Duffield *et al.* (90.01 μg/L) [7]. The yellow line 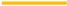, indicates concentration required to optimise plasma GPx activity according to the WHO (63.16 μg/L) [61].

Overall, the results indicate that if 98.7 μg/L of Se in plasma or serum are required to optimize GPx activity [8], then suboptimal Se status is found in both regions, but is less consistent in the Middle East. Figure 2 shows the serum and/or plasma Se concentrations of study subjects in various European countries (including the UK) in relation to the suggested levels required for full GPx expression, as well as the level proposed by the WHO to achieve two thirds of the GPx expression. Figure 3 gives the same information in relation to the Se concentrations found for the study subjects in various Middle Eastern countries.

#### 3.2.1. Selenium Status Studies: European Countries/UK

Results of Se status studies in Europe suggest suboptimal blood Se concentrations for populations in most of this region if 90.01 μg of Se/L of plasma or serum is the assumed requirement for full plasma GPx expression [7]. Exceptions were Austria [89], Hungary [98], healthy subjects in Denmark [75,95], healthy subjects, but not obese [86] or those with Type 1 diabetes [87] in Poland and some of the participants in the Dietary Habit Profile in European Communities with Different Risk of Myocardial Infarction: the Impact of Migration as a Model of Gene-Environment Interaction “IMMIDIET” study [84]. The lowest reported serum Se status of all European studies investigated was in Albanian adults living in Greece, with 37.4 μg/L [62]. Schulpis *et al.* [62] speculated these low values could be related to their poor animal protein intake, which could be the consequence of their low socioeconomic status. The highest Se status of all studies in the European region was in Poland, where boys had a mean serum Se concentration of 111.1 μg/L [86]. Finland was an exception, where Se levels were amongst the lowest in the world until the early 1980s, before the implementation of a nationwide Se fertilisation programme. People in this region are now Se sufficient [83,90].

There were unusual findings noted in some studies from European countries. For example, there was a larger than expected gender effect in plasma Se concentrations reported from Granada, Spain, where males had 87 μg/L and women had 67 μg/L [96]. There was no explanation for this gender difference in the reference, and lifestyle factors, including smoking, alcohol consumption, *etc.*, were reported to show no significant association with plasma Se levels. However, there were significantly higher plasma Se levels in individuals with higher energy intake who performed more physical activity, although this was not reported separately by gender [96]. Therefore, it is difficult to speculate the reason for the gender discrepancy.

UK studies, including a broad range of population groups, identified a fairly consistent overall suboptimal Se status in this country based on the range of 90–100 μg/L to achieve maximum GPx expression [20,37,60,70,71,77,78,79,80,81]. Pre-term infants in the Hampshire area who had received parenteral nutrition and blood transfusions had significantly lower mean plasma Se levels that normalised by six months of gestation-corrected age. However, term infants had normal Se status at birth and six months of age [70]. In 14–18-year-old adolescents where plasma Se was determined between 28 and 32 weeks of gestation, plasma Se concentration was lower in those who gave birth to small—for gestational age (SGA) infants compared to those having appropriate-for-gestational age (AGA) infants [79]. Smoking mothers had a lower Se concentration compared with non-smokers, and Afro-Caribbean women had higher Se concentrations compared with white Europeans [79]. The overall low plasma Se concentration in these adolescent mothers was speculated to contribute to the risk of delivering an SGA infant, possibly through lowering placental antioxidant defence, thus directly affecting foetal growth [79]. Differences in plasma Se between ethnicities was thought to be related to nutritional intake variation [79]. Longitudinal analysis of healthy British middle-aged adults found suboptimal Se status in both males and females [37]. Analysis of plasma/serum Se concentration in a number of D-FINES study participants showed that full plasma GPx expression (serum or plasma Se level of 98.7 μg/L) was not achieved in the majority of all population groups included in the study, and postmenopausal women had higher Se status across all ethnicities [24]. Se deficiency in independent and institutionalised adults over age 65 years was also consistent and found to be lowest in the autumn and highest in spring [71]. However, a study investigating the effect of Se supplementation on thyroid function in the elderly found a mean baseline plasma Se concentration sufficient to achieve maximum GPx expression [5].

#### 3.2.2. Selenium Status Studies: Middle Eastern Countries

Se status studies throughout the Middle East provided varying results. Even within the same country, this variability was apparent. For example, in Iran, mostly case-control studies reported serum Se concentrations below 90 μg/L [106,109,110,123,128,129,130], almost as many reporting serum Se content between 90 and 100 μg/L [105,107,108,127], and some found above 120 μg/L [111,124,125]. The highest serum Se in normal/control subjects was found in Lebanon (151.2 μg/L) [112], and the lowest was reported in Turkey in school children from an endemic goitre area (31 ± 23 μg/L) [45].

The majority of studies from Turkey indicated that the population had suboptimal Se status [23,45,103,104,132,133,135] if 90.01 μg of Se/L of plasma or serum is the assumed requirement for full plasma GPx expression [7]. However, two studies reported plasma [134] and serum [136] Se concentrations slightly above 120 μg/L.

All of the case-control studies from Egypt reported serum Se content of less than 90 μg/L in healthy control subjects [137,138,139].

Analysis of serum/plasma samples from the Saudi Arabian Bone Health Study, funded by King Abdulaziz University, showed that a large proportion of the KSA women under investigation had low Se status [24]. High blood Se concentrations were recorded in some KSA studies, including patients with cardiovascular disease [1,118], in a Lebanon study investigating metabolic syndrome [112] and one study in Jordan investigating Se status in smokers and non-smokers [114].

Urinary Se levels were investigated in 170 KSA diabetics and controls. Mean (SEM) urinary Se concentration was 0.394 (0.017) μM or (31.1 (1.3) μg/L) and mean (SEM) urinary Se/mol creatine concentration was 56 (2.9) μM or (39.1 (2) μg/g creatine) [119].

Toenail analysis, which is a measure of intake over a longer term, indicates low consumption in the Al-Kharj district [21,102]. Although none of the participants had serum Se levels below 45 μg/L, which would be considered as inadequate, 41% had toenail Se below 56 μg/g, indicative of a low Se status. A 2006 study that reported serum and toenail Se status indicated a similar outcome in children from the same district. In that study, serum and toenail Se were significantly related, a finding that could reflect dietary Se intake [116].

Infant blood Se status, according to more than one study in the Middle East, is within the normal range [103,115], while one study in Yemeni infants found Se blood status to be very low [113]. However, studies that date back to the 1990s indicate low Se content in breast milk [52]. One study of Kuwaiti and non-Kuwaiti women residents in Kuwait indicates that the latter have Se breast milk levels below the 18.5 μg/L (international reference range) at term and from 6 to 12 months during the lactation period [61]. Kuwaiti women had sufficient levels at term, but dropped just below the recommended level from 6 to 12 months. These results agree with low maternal-foetal Se status found in gestational diabetic pregnancies, where serum Se concentrations for the obese control and diabetic groups were below the level required to optimise GPx activity [117].

## 4. Discussion

This review of recently published studies on Se intake and status throughout Europe, the UK and the Middle East shows that Se deficiency is widespread among these populations and agrees with previous reports highlighting the problem. An earlier report of low serum or plasma Se throughout the UK population included data from the UK PRECISE study [78], where the mean plasma Se at baseline was 90.8 μg/L, below the Se concentration required for optimal plasma GPx activity according to Alfthan *et al.* [8]. Another publication reported daily Se intake less than the RDA of 55 μg for numerous countries throughout Europe (including the UK) and the Middle East [3].

The low Se intakes in the UK population, as reported in the NDNS 2008/2009 [36] and mirrored in the D-FINES study [20,60], agree with previous government surveys, including that of the Ministry of Agriculture, Fisheries and Foods and an analysis of Se in food completed during the mid to late 1990s [142], where UK Se intakes ranged from 29 to 39 μg/day [19]. Given the geographical location of Northern Ireland relative to the UK, it is not surprising that the Se intake is less than the RDA in that country, as well [38].

The Se blood concentrations reported in the various studies outlined in this review indicate low to borderline Se status in the participants if 90.01 μg of Se/L of plasma or serum is the assumed requirement for full plasma GPx expression [7]. However, if the higher Se concentration of 98.7 μg/L were required [8], then the Se status of many study subjects would be considered inadequate. As plasma and serum Se concentration only reflects Se intake over the short term, it is unclear what similar long-term Se measurements would show. Bearing in mind that a high proportion of the UK population fail to obtain the RNI for Se and with Se soil levels low in the UK, it is highly likely that the Se status of the UK population is less than adequate. It appears that in spite of acknowledging the risk of low Se intakes in the UK population over a decade ago, little impact has been made to improve this situation [36]. A switch from importing wheat grown within the United States and Canada to that grown in Europe is believed partly to blame for the low dietary Se intake in recent years. However, even if optimal plasma Se levels (e.g., 105–115 μg/L, compared with a current estimated mean of approximately 87 μg/L) were attained within the UK population, there is no guarantee that population disease levels would improve in the medium term. Although, some individuals, such as heavy smokers or people with specific mutations within genes coding for mitochondrial antioxidant enzymes [10], would likely benefit with increased Se intake.

Se status/intake studies within the overall Middle East provide varying results. Toenail analysis indicates low consumption in the Al-Kharj district. Serum Se levels are regarded to reflect short-term Se intakes, whereas the toenail provides a better measure for long-term Se intakes [6]. This finding concurs with the low Se intake levels in infants and babies of between 0.9 and 15 μg/day [52]. It is interesting to note that in certain areas of Jordan, the Se content of aquifers is very high, which might have had an influence on the very high blood Se levels found in smokers and non-smokers in that country. In addition, Se content from tobacco products might contribute to the even higher blood Se concentrations found in smokers. In Turkey and Iran, where many studies have been carried out to assess Se status in different population and patient groups, some studies reported very low blood Se levels, similar to those in Eastern Europe; a few were high, but overall, the values were comparable to Central European status. If a serum or plasma Se concentration of 90.01 μg/L is the assumed level for sufficiency, then more of the reviewed Middle Eastern studies would meet this reference value than not.

The KSA studies indicated that the soil Se levels and food grown in those areas within KSA [48] are low to deficient. Similar variation in Se content has been reported in Hungary [98], which is considered a low to deficient Se area in Europe according to current Se intake recommendations [15]. There is currently an effort by the KSA government to ensure food security in the Kingdom by encouraging home-grown produce. However, food imports form a significant part of the food supply, with a large part of this sourced from the United States, where soil Se levels tend to be high to adequate [15,143]. This could be contributing to higher Se intake in some people in KSA and may be reflected in the King Abdulaziz University study in Jeddah [49]. In this study, the major food groups providing Se were cereals/cereal products, legumes and meats. Infant formulas imported from Europe may ensure adequate intake in infants consuming those nutrition sources [50,51]. However, some breast-fed children may not be obtaining sufficient Se [51,52]. Dietary habits may differ between rural and urban areas and different parts of the country, where the availability of certain imported food items might differ. This, in addition to food security influences and social status, could have a considerable effect on Se status and explain the varied Se concentrations that were found in the KSA population.

## 5. Conclusions

Overall, the results of this systematic review indicate that Se intake and status is suboptimal in European and Middle Eastern countries with less consistency in the Middle East according to current measures of sufficiency. These results combined with growing knowledge of the importance of Se to overall health warrant more work initially to establish an agreed range of blood Se concentration or other markers to determine optimal Se intake and, subsequently, to ensure adequate Se supplementation in populations at risk of low Se intake. The Se fertilisation programme in Finland would appear to have successfully raised Se status in that population.
